# Single-Walled Carbon Nanotube (SWCNT)-induced interstitial fibrosis in the lungs of rats is associated with increased levels of PDGF mRNA and the formation of unique intercellular carbon structures that bridge alveolar macrophages *In Situ*

**DOI:** 10.1186/1743-8977-3-15

**Published:** 2006-11-29

**Authors:** James B Mangum, Elizabeth A Turpin, Aurita Antao-Menezes, Mark F Cesta, Edilberto Bermudez, James C Bonner

**Affiliations:** 1 From CIIT Centers for Health Research, Research Triangle Park, North Carolina 27709, USA

## Abstract

**Background:**

Nanotechnology is a rapidly advancing industry with many new products already available to the public. Therefore, it is essential to gain an understanding of the possible health risks associated with exposure to nanomaterials and to identify biomarkers of exposure. In this study, we investigated the fibrogenic potential of SWCNT synthesized by chemical vapor deposition using cobalt (Co) and molybdenum (Mo) as catalysts. Following a single oropharyngeal aspiration of SWCNT in rats, we evaluated lung histopathology, cell proliferation, and growth factor mRNAs at 1 and 21 days post-exposure. Comparisons were made to vehicle alone (saline containing a biocompatible nonionic surfactant), inert carbon black (CB) nanoparticles, or vanadium pentoxide (V_2_O_5_) as a known inducer of fibrosis.

**Results:**

SWCNT or CB caused no overt inflammatory response at 1 or 21 days post-exposure as determined by histopathology and evaluation of cells (>95% macrophages) in bronchoalveolar lavage (BAL) fluid. However, SWCNT induced the formation of small, focal interstitial fibrotic lesions within the alveolar region of the lung at 21 days. A small fraction of alveolar macrophages harvested by BAL from the lungs of SWCNT-exposed rats at 21 days were bridged by unique intercellular carbon structures that extended into the cytoplasm of each macrophage. These "carbon bridge" structures between macrophages were also observed *in situ *in the lungs of SWCNT-exposed rats. No carbon bridges were observed in CB-exposed rats. SWCNT caused cell proliferation only at sites of fibrotic lesion formation as measured by bromodeoxyuridine uptake into alveolar cells. SWCNT increased platelet-derived growth factor (PDGF)-A, PDGF-B, and PDGF-C mRNA levels significantly at 1 day as measured by Taqman quantitative real-time RT-PCR. At 21 days, SWCNT did not increase any mRNAs evaluated, while V_2_O_5 _significantly increased mRNAs encoding PDGF-A, -B, and -C chains, PDGF-Rα, osteopontin (OPN), connective tissue growth factor (CTGF), and transforming growth factor (TGF)-β1.

**Conclusion:**

Our findings indicate that SWCNT do not cause lung inflammation and yet induce the formation of small, focal interstital fibrotic lesioins in the alveolar region of the lungs of rats. Of greatest interest was the discovery of unique intercellular carbon structures composed of SWCNT that bridged lung macrophages. These "carbon bridges" offer a novel and easily identifiable biomarker of exposure.

## Background

The commercial interest in single walled carbon nanotubes (SWCNT) is rapidly increasing due to their superior mechanical, electric and thermal properties [1]. With the development of mass production and handling facilities for SWCNT, it is critical to fully understand the risk associated with exposure since it is likely that increasing human exposure will occur [2]. Moreover, it would be highly desirable to identify specific biomarkers of SWCNT exposure in order to identify individuals who are exposed occupationally or consumers who become exposed to products containing SWCNT. Individual SWCNT are less than 2 nm in diameter and form fibrous ropes that range up to several microns in length [3]. Ropes of SWCNT further aggregate by means of electrostatic charge to form larger, micron-sized agglomerates.

Several recent studies indicate fibrogenic effects of SWCNT instilled into the lungs of mice or rats. Lam et al reported that SWCNT instilled into the lungs of mice caused the formation of fibroproliferative lesions to the same extent as toxic quartz particles [4]. In a similar study, Warheit and colleagues reported that SWCNT exposure in rats by intratracheal instillation caused pulmonary granulomas, although there was a lack of lung toxicity as assessed by lavage parameters and cell proliferation parameters [5]. Shvedova and coworkers reported that pharyngeal aspiration of SWCNT in mice caused progressive intersitital fibrosis and granulmonas, along with increased levels of cytokines (IL-1β and TNF-α) within 1 day and elevated TGF-β1 after 1 week [6]. All of these studies indicate that SWCNT cause some lung toxicity when instilled or aspirated as a bolus of agglomerated nanoropes. Nevertheless, it remains unclear from these reports whether SWCNT have the potential to stimulate pulmonary fibrogenesis under conditions of occupational or environmental exposure.

The overall goal of this study was to investigate changes in the expression of genes encoding known profibrogenic mediators *in vivo *following SWCNT exposure and correlate gene expression patterns with the formation of fibrotic lesions in the lung. Surprisingly, no inflammation was observed in SWCNT-exposed rats, although focal interstitial fibrotic lesions were observed near clusters of macrophages containing micron-sized aggregates of SWCNT within the alveolar region of the lung. Moreover, small but significant increases in lung mRNAs encoding three PDGF ligands (PDGF-A, -B, -C) were observed at 1 day post-SWCNT exposure but were not elevated in the lungs of rats exposed to carbon black (CB) nanoparticles with a similar size and specific surface area. Interestingly, SWCNT formed unique intercellular structures that bridged alveolar macrophages *in situ*. These structures were not observed in the lungs of CB-exposed rats. While our results indicate relatively weak fibrogenic potential of SWCNT in the lungs of rats, carbon bridge structures between macrophages allowed easy identification of animals exposed to SWCNT as compared to those exposed to CB. To our knowledge this is the first report of a biomarker of carbon nanotube exposure.

## Results

### SWCNT stimulate the formation of focal interstitial fibrotic lesions in the lungs of rats

**Figure 1:**
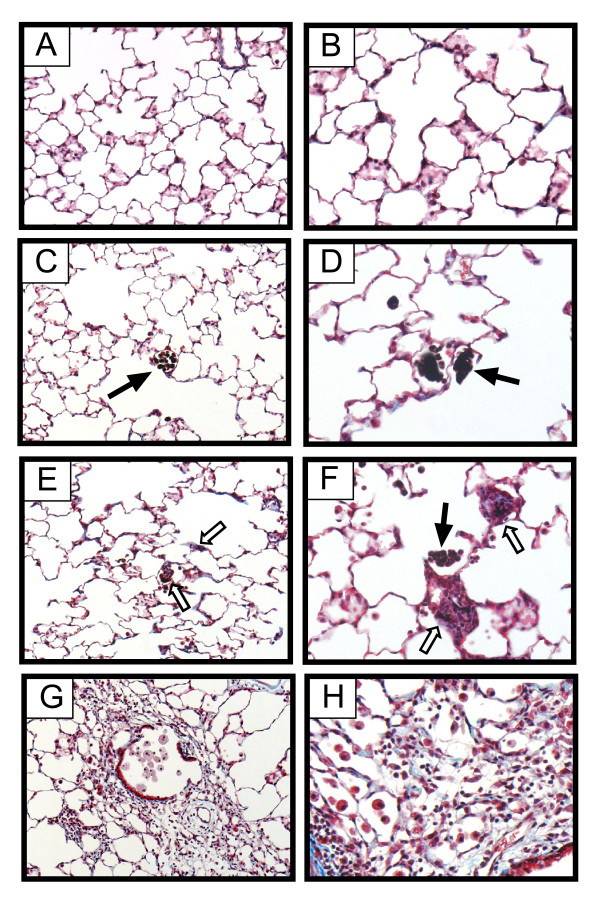
Lung histopathology from rats 21 days after a single oropharyngeal aspiration exposure to saline vehicle containing 0.1% Pluronic F-68 (**A, B**), carbon black ultrafine particles (**C, D**), aggregates of single walled carbon nanotubes, SWCNT (**E, F**), or vanadium pentoxide (V_2_O_5_) (**G, H**). Carbon inclusions are indicated by closed arrows in panels C, D, and F. Regions of alveolar wall thickening in the lungs of rats exposed to SWCNT are indicated by open arrows adjacent to carbon-filled macrophages in panesl E and F. Original magnification 20× (panels A, C, E, G) or 40× (panels B, D, F, H).

Clusters of carbon-laden macrophages were observed in the lungs of either CB or SWCNT-exposed rats after 21 days (Fig. 1). No fibrotic lesions were observed in the lungs of CB-exposed rats (Fig. 1C,D). Relatively small trichrome-positive fibrotic lesions (~100 μm diameter) were observed within the alveolar regions of SWCNT-exposed rats and were adjacent to clusters of alveolar macrophages that contained carbonaceous materials (Fig. 1E,F). These fibrotic foci were sparsely distributed within the alveolar region of the lung and affected less than 5% of the total lung tissue. LDH and total protein levels measured in BALF collected from SWCNT-exposed rats were not statistically changed from controls or CB groups (data not shown). V_2_O_5 _caused the formation of fibrotic lesions in the alveolar region and around airways (Fig. 1G,H), consistent with our previous observation of V_2_O_5_-induced lung injury in rats [7].

### Intercellular carbon structures that bridge alveolar macrophages are readily identifiable in cytospins of BAL from SWCNT exposed rats

**Figure 2:**
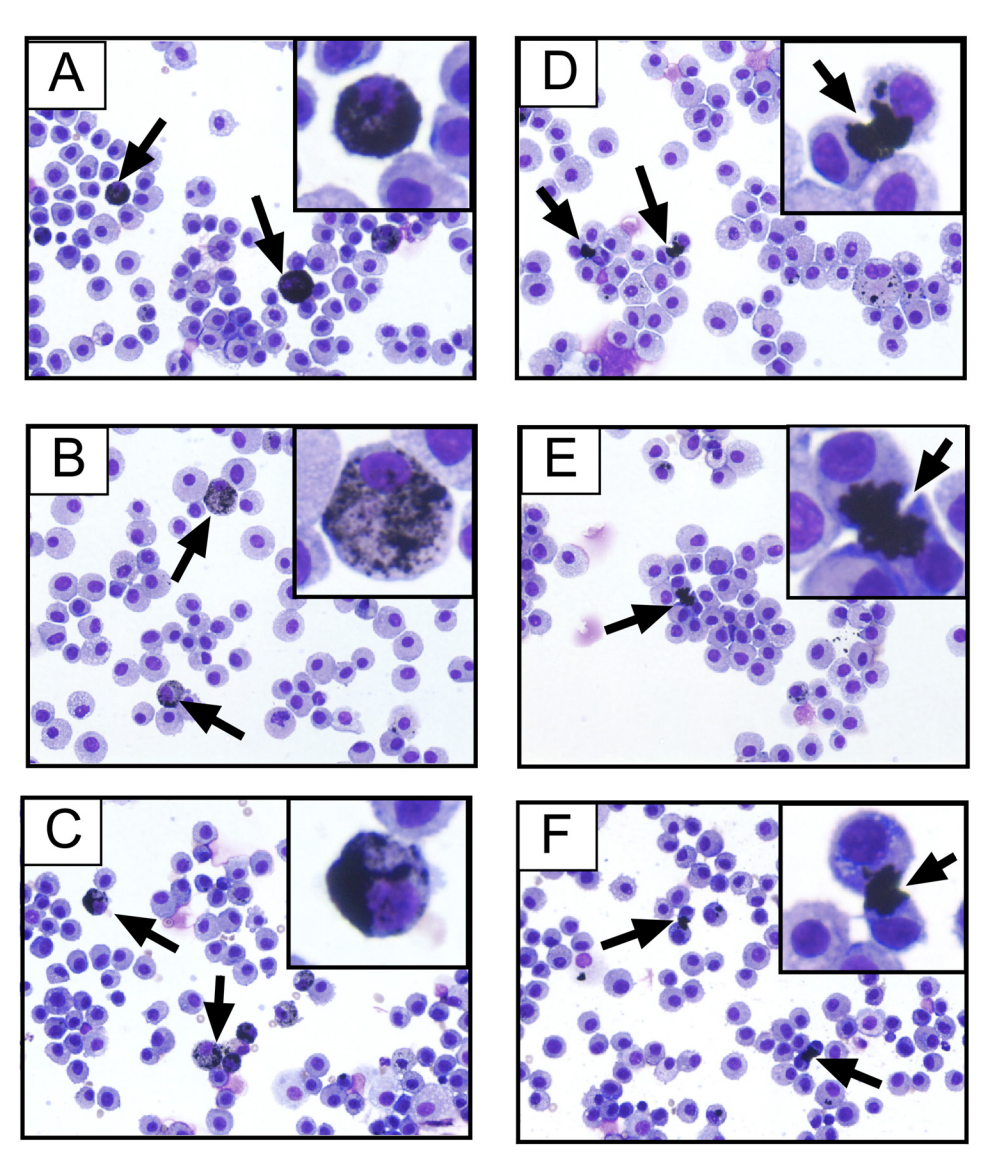
Cytospins of bronchoalveolar lavage (BAL) cells from rats exposed to for 21 days to carbon black ultrafine particles (**A–C**) or single wall carbon nanotubes, SWCNT (**D–F**). Carbon inclusions are indicated by arrows. Carbon bridges between macrophages formed only in SWCNT-exposed rats. Original magnification (40×), inset (80×).

**Figure 3:**
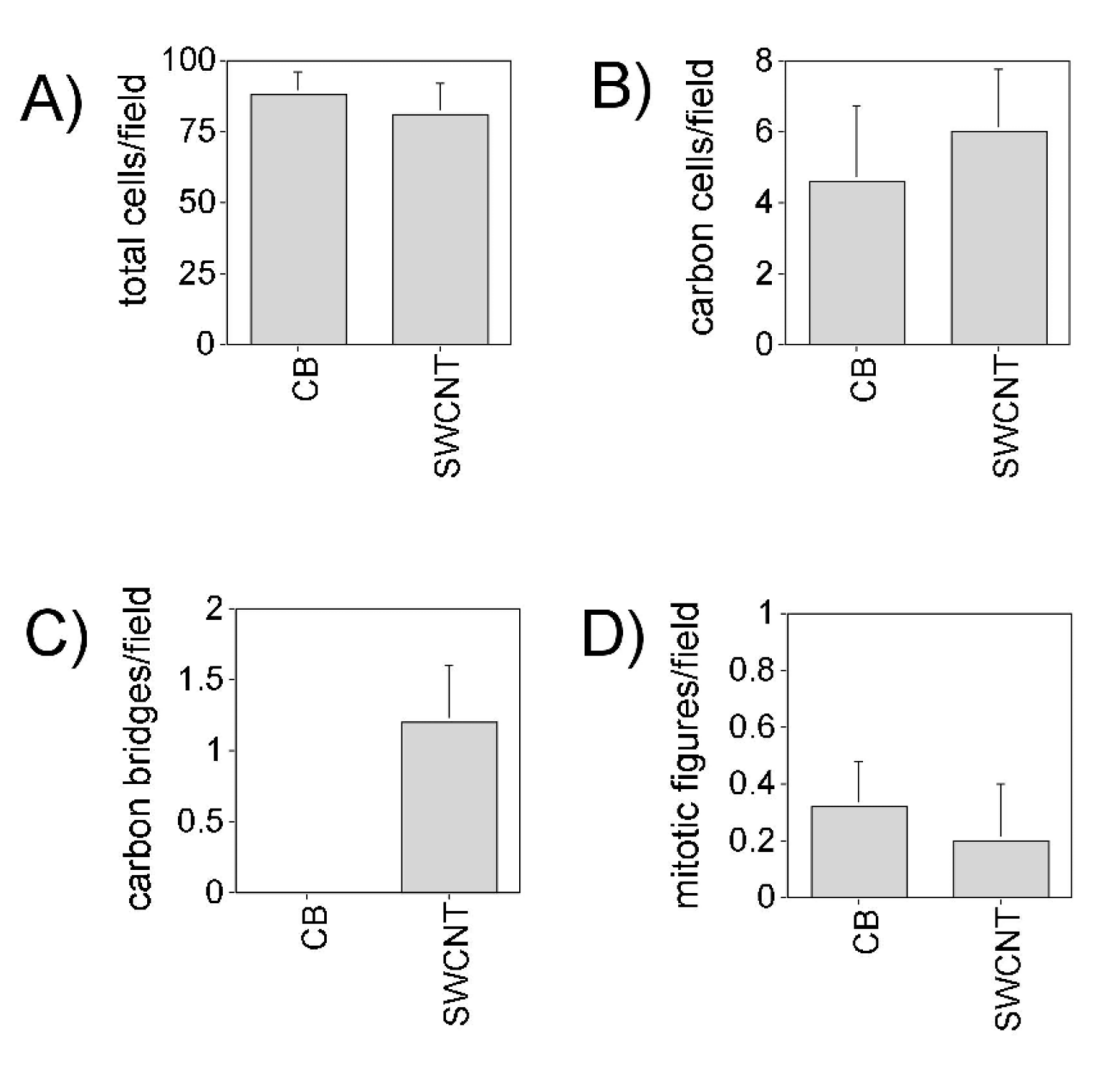
Quantitation of BAL cells in cytospins for assessment of carbon bridge formation between macrophages from 21 day SWCNT-exposed or carbon black (CB)-exposed rats. Each bar represents the mean ± SD of five animals. Ten fields were counted per animal and at least 500 cells per animal were counted. (**A**) Total cells/field, (**B**) Numbers of cells per field containing carbon inclusions, (**C**) Carbon bridges per field (only found in SWCNT-exposed rats), and (**D**) Mitotic figures per field.

Differential analysis of BAL cell cytospins from CB or SWCNT-exposed rats showed no overt inflammatory response, with greater than 95% macrophages and the remaining 5% or less comprised of neutrophils or lymphocytes. A small percentage of macrophages in either the CB or SWCNT groups contained carbon inclusions (Fig. 2). An even smaller number of BAL macrophages from the SWCNT-exposed rats were linked by readily identifiable carbon bridges at 21 days post-exposure. These carbon bridges composed of SWCNT were ~5 μm in length and possessed a unique appearance characterized by fanned ends extending into the cytoplasm of each macrophage, giving the overall appearance of an hourglass (Fig. 2D–F). These structures were not apparent in BAL cytospins from rats treated for 1 day with SWCNT. Although some macrophages were joined by SWCNT aggregates at day 1, these were not the well-formed hourglass structures that were readily identifiable at 21 days. Carbon bridges were not observed in BAL cells isolated from CB-exposed rats (Fig. 2A–C). The results of the cytology and carbon bridge assessment are shown in Fig. 3. There were no differences in the total numbers of macrophages per field between CB and SWCNT-exposed groups (Fig. 3A). In both CB and SWCNT groups, approximately 5% of the total cells per field contained carbon inclusions of a similar size range (0.1 to 5 μm in diameter) (Fig. 3B). In the SWCNT groups only, approximately one fourth of the carbon inclusions within cells were identified as carbon bridges (Fig. 3C). No significant differences were observed in the numbers of mitotic figures, i.e., macrophages undergoing cell division, between CB and SWCNT groups (Fig. 3D).

### SWCNT induce cell proliferation in small granulomatous lesions and cause carbon bridge formation between macrophages in situ

**Figure 4:**
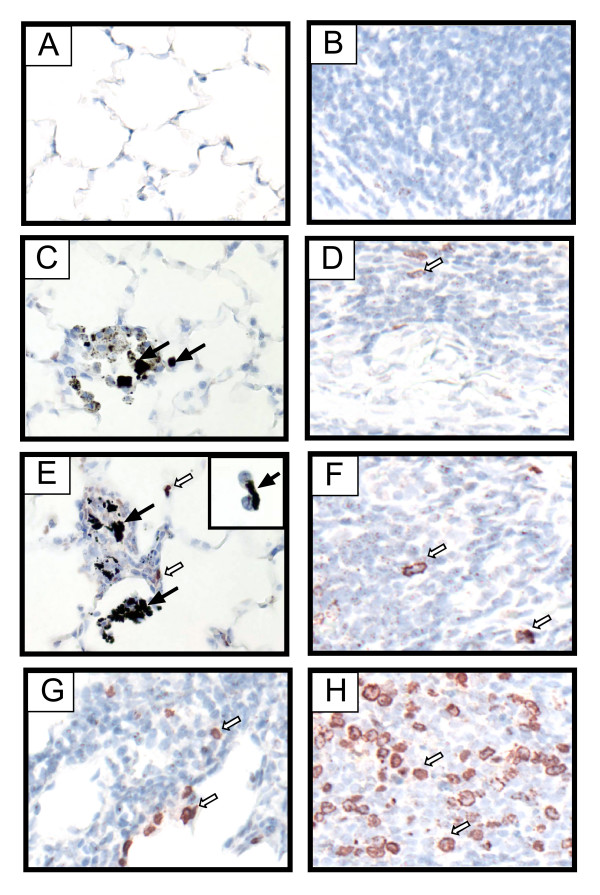
Bromodeoxyuridine (BrdU) immunohistochemistry showing a cell proliferative responses 21 days post-exposure in the lung alveolar region (panels **A, C, E, G**) and in the bronchus-associated lymphoid tissue (panels **B, D, F, H**). Saline vehicle containing 0.1% Pluronic F-68. (**A, B**). Carbon black treatment (**C, D**). SWCNT treatment (**E, F**). Vanadium pentoxide (V_2_O_5_) treatment (**G, H**). Solid arrows in panels C and E indicate aggregates of carbon black and SWCNT, respectively. Inset shows two macrophages joined by a carbon bridge within a nearby alveolar space. Open arrows indicate representative BrdU-positive nuclei. Original magnification for all panels, 40×.

**Figure 5:**
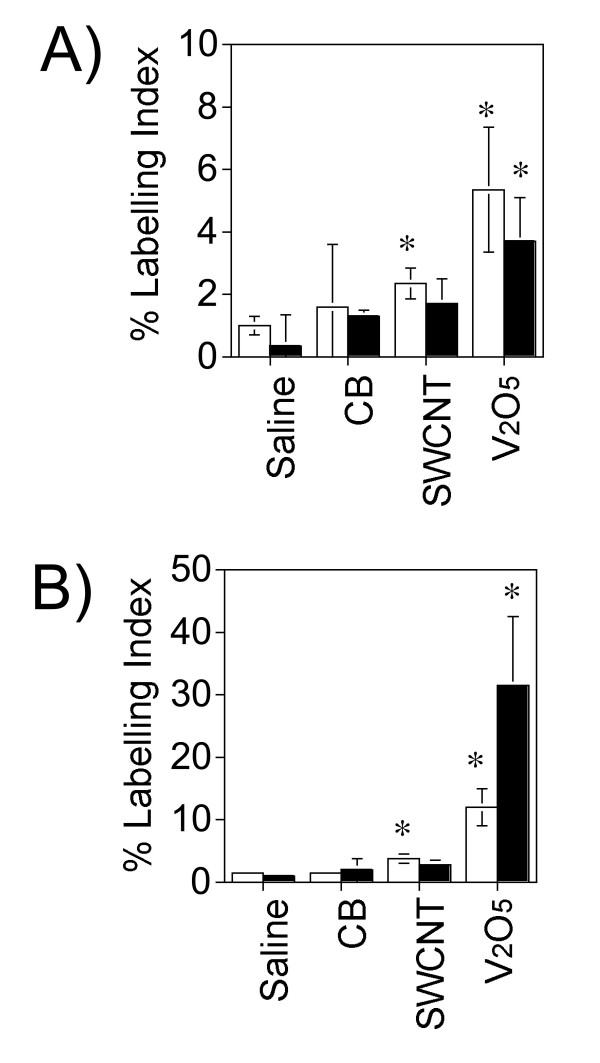
Cell proliferation at (**A**) day 1 and (**B**) day 21 post-exposure in the lungs of mice exposed by oropharyngeal aspiration to saline vehicle containing 0.1% Pluronic F-68, carbon black (CB), single-walled carbon nanotubes (SWCNT) or vanadium pentoxide (V_2_O_5_). Open bars represent the labeling index in the alveolar region of the lung (open bars) and solid bars represent the labeling index in the bronchus-associated lymphatic tissue. Labelling indices were measured as described in Methods and are reported as the percentage of BrdU-labeled cells of the cells counted (minimum of 400 cells counted). *P < 0.05 compared to saline control.

The light hematoxylin counterstain used for BrdU immunohistochemistry revealed a wide distribution of CB and SWCNT aggregates possessing a similar size range of 0.5 to 5 μm in diameter within the alveolar region. A background labeling index of ~1% BrdU-positive cells were present in the alveolar region or bronchus-associated lymphatic tissue (BALT) of saline vehicle-exposed control rats (Fig. 4A,B and Fig. 5). No significant increase in BrdU incorporation was observed in the alveolar region of CB-exposed rats, although carbon-containing macrophages were sparsely distributed within the alveolar region and were observed clustered in alveolar spaces (Fig. 4C). SWCNT-exposed rats contained BrdU-positive interstitial cells and epithelial cells in the alveolar region and these were limited to sparse fibroproliferative lesions (Fig. 4E). A small number of alveolar macrophages joined by carbon bridges were observed *in situ *within the alveolar region of SWCNT-exposed rats (Fig. 4E, inset panel). These *in situ *carbon bridges between macrophages were not observed in the lungs of CB-exposed rats. SWCNT caused a significant increase in the BrdU labeling index at both day 1 and day 21 (Fig. 5). SWCNT and CB caused a slight but not statistically significant increase in lymphocytes within BALT at day 1 or day 21 (Fig. 4D,F and Fig. 5). V_2_O_5 _caused a significant increase in the BrdU labeling index in the alveolar region and particularly within BALT at day 1 and day 21 (Fig. 4G,H and Fig. 5).

### Profibrotic growth factors and procollagen gene expression in lungs from CB- and SWCNT-exposed rats

**Figure 6:**
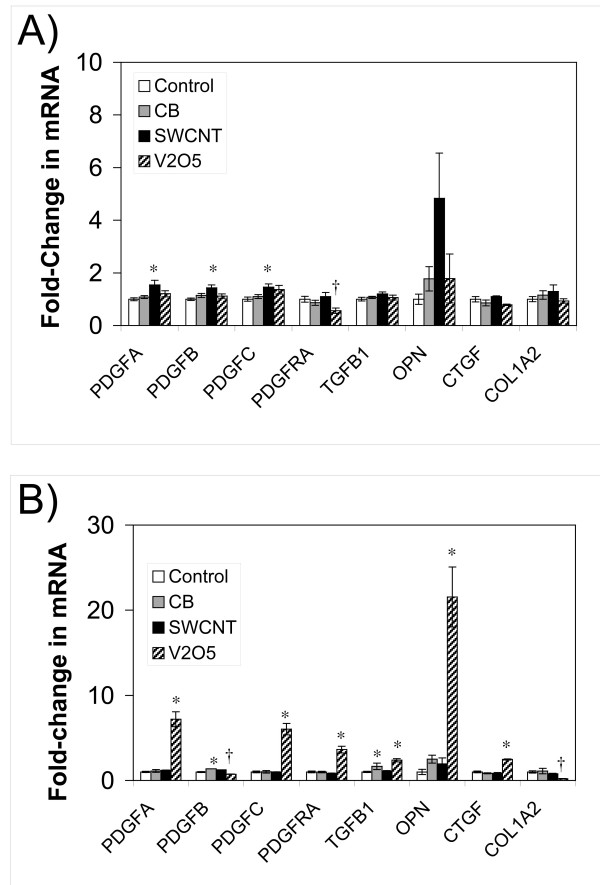
Levels of growth factor and procollagen mRNAs in the lungs of rats exposed to SWCNT, CB, or V_2_O_5_. Whole lung RNA was extracted as described in Methods and mRNA expression measured by quantitative real-time RT-PCR. V_2_O_5 _was used as a positive control for inducing mRNA levels of profibrogenic mediators. (**A**) 1 day post-exposure. (**B**) 21 days post-exposure. Control animals were treated with vehicle alone (PBS with 0.1% Pluronic F-68). Data are presented as mean values ± SEM from three to five animals per group and expressed as the fold-change in mRNA relative to an 18S rRNA housekeeping gene. *P < 0.05 significantly increased or †P < 0.05 significantly decreased compared to control.

Quantitative real-time RT-PCR was performed on whole lung mRNA from CB, SWCNT, and V_2_O_5_-exposed rats to measure changes in the gene expression of a variety of profibrotic growth factors or procollagen (COL1A2). Platelet-derived growth factor (PDGF-A, -B, and -C) mRNA levels were significantly increased in SWCNT-exposed rats at day 1, although less than 2-fold over saline controls (Fig. 6A). SWCNT also increased OPN mRNA levels day 1(~5-fold) and day 21 (~2-fold) although neither was statistically significant. V_2_O_5 _caused a significant increase in PDGF-A, -B and -C, PDGF receptor-alpha (PDGF-Rα), transforming growth factor-β1 (TGF-β1), OPN, and connective tissue growth factor (CTGF) at 21 days post-exposure. Procollagen mRNAs were not induced by either CB or SWCNT, but were decreased 5-fold by V_2_O_5 _(Fig. 6B).

## Discussion

SWCNT have been reported to cause the formation of granulomas, a type of pulmonary fibrotic reaction in the lung. The progression of granulomas in the lung interstitium is mediated in large part by alveolar macrophages, which accumulate at sites of particle deposition and become activated by particle phagocytosis [8,9]. Once activated, macrophages produce polypeptide growth factors that stimulate the proliferation of interstitial fibroblasts, which are the principal collagen-producing cell that drives fibrogenesis [10]. In the present study, we observed small, interstitial fibrotic lesions in the lungs of rats 21 days after exposure to SWCNT. These fibroproliferative lesions were associated with clusters of alveolar macrophages containing carbonaceous material. CB nanoparticles of similar size and specific surface area also caused focal clustering of macrophages with carbon inclusions but did not induce fibrotic lesions. Our findings extend the work of others in that we report 1) the induction of lung mRNAs encoding PDGF ligands after SWCNT exposure, and 2) the formation of intercellular carbon bridge structures between alveolar macrophages in the lungs of SWCNT-exposed rats.

Key growth factors that stimulate fibroblasts to proliferate and/or migrate to sites of lung injury are PDGF [11], CTGF [12], and OPN [13]. Other growth factors such as TNF-α and TGF-β1 stimulate fibroblasts to deposit a collagen matrix, which defines a fibrotic lesion [14]. We found that SWCNT induced small but significant increases in the levels of mRNAs encoding three PDGF ligands (PDGF-A, -B, and -C) at day 1 post-exposure. SWCNT also increased OPN mRNA levels ~5-fold at day 1 post-exposure. At 21 days after SWCNT exposure we observed slight increases PDGF-B and OPN, whereas V_2_O_5 _induced robust and significant increases in mRNAs encoding PDGF-A, PDGF-C, PDGFRα, TGF-β1, OPN, and CTGF. In particular, the induction of PDGFRα by V_2_O_5 _confirmed our earlier observation that this growth factor receptor is up-regulated *in vivo *during fibrogenesis [7]. In general, the induction of mRNA levels for profibrogenic factors by V_2_O_5 _21 days post-exposure correlated with the formation of diffuse interstitial and airway fibrotic lesions within the lung. Moreover, the induction of mRNAs encoding PDGFs and OPN by SWCNT at day 1 correlated with the formation of small, focal fibrotic lesions at day 21. In contrast, CB nanoparticles caused neither induction of genes nor fibrotic lesions. The increase in mRNA encoding PDGF family members observed at day 1 after SWCNT exposure suggest that PDGF could play a role in the formation of SWCNT-induced fibrotic lesions as PDGF and its receptors are known to mediate fibroblast chemotaxis and proliferation during fibrogenesis [11].

In agreement with other studies of SWCNT instillation or pharyngeal aspiration in mice or rats, we observed interstitial fibrotic lesions, albeit these lesions were relatively small (~100 μm) and sparsely distributed. Other investigators have reported more severe fibrotic or granulomatous lesions within the lungs of mice or rats after SWCNT exposure. Differences between the toxicity of SWCNT in this study as compared to some earlier studies could be due to differences in the delivered dose of aggregated SWCNT or differences in the size of SWCNT aggregates delivered to the distal lung. We aspirated 2 mg/kg SWCNT into the lungs rats in our experiments, whereas others have used concentrations that range from 4 to 20 mg/kg [4,5]. Shvedova and colleagues used a dose of SWCNT comparable to the present study and reported significant progressive interstitial fibrosis at deposition sites of more dispersed nanostructures [6]. While we observed most fibrotic lesions closely adjacent to macrophage clusters and micron-sized SWCNT aggregates, we also observed interstitial thickening within alveolar walls characterized by increased trichrome staining at sites distant from SWCNT aggregates and macrophages. While our study did not show lesions as severe as those reported by Shvedova, there were several potentially important differences in our materials. First, our SWCNT were synthesized by a different method (chemical vapor deposition) and contained different metal catalysts (cobalt and molybdenum). The SWCNT used by Shvedova and coworkers contained trace levels of iron catalyst. Second, while we used similar suspension concentrations of SWCNT to those used by Shvedova et al (i.e., 2 mg/kg), based on histopathologic evaluation it is clear that we did not achieve the same efficiency of delivery. Finally, we dispersed our SWCNT with nonionic surfactant that could have decreased the bioactivity of nanoparticles. Finally, we exposed rats to SWCNT, while Shvedova and colleagues used mice.

The dose of SWCNT used in our rodent exposures is presumably higher than would be encountered in human exposure situations assuming that the delivered dose reached the lung. Estimates of airborne concentration of nanotubes material generated during handling are below 53 μg/m^3 ^[3]. However, a limitation of oropharyngeal aspiration and intratracheal instillation techniques is that a bolus of particles is delivered in suspension and may not be evenly distributed within the lung or may inadvertently pass into the esophagus and swallowed. This could significantly reduce the predicted dose to the distal lungs. A far superior technique to deliver nanoparticles and nanotubes is inhalation exposure, especially for SWCNT that are known to have a high electrostatic potential and are therefore predisposed to agglomeration. However, even when aerosolizing SWCNT for inhalation exposure, micro-sized particles are formed that will likely deposit in the lung according to the principles of sedimentation rather than diffusion. Nevertheless, inhalation studies will no doubt be invaluable in addressing health effects of nanomaterials.

A potentially important factor in determining the toxicity of carbon nanotubes is the presence of contaminating metals that were used as catalysts in the manufacturing process. While much emphasis has been given to particle size, particle shape, and increased specific surface area as a determinant of particle toxicity, our study showed that 8 nm diameter carbon black (CB) nanoparticles (composed entirely of elemental carbon) caused no inflammation or fibrosis in the lungs of rats, although macrophages accumulated at sites of CB deposition in the lung and engulfed these nanoparticle aggregates. The SWCNT and CB used in this study possessed similar specific surface areas (~300 to 600 m^2^/g) and yet at equivalent doses of CB and SWCNT, only the SWCNT stimulated fibrotic responses at day 21 post-exposure. Therefore, the fibrogenic activity of SWCNT in comparison to CB is likely due to either differences in shape or elemental composition. The SWCNT used in this study were synthesized by chemical vapor deposition using cobalt and molybdenum as catalysts. While the raw nanotubes formed by this process are subsequently acid washed to remove contaminating metal catalysts, residual cobalt and molybdenum remained as 2.6% and 1.7% of the total elemental composition, respectively. Individuals exposed to occupationally to cobalt are at risk of developing "hard metal disease", which includes the formation of interstitial pulmonary fibrotic lesions [15]. Other reports of SWCNT-induced interstitial fibrosis have used materials synthesized by laser ablation or a high-pressure carbon monoxide disproportionation (HiPCO) process, both of which require metal catalysts [4-6]. Warheit and colleagues used SWCNT synthesized by laser ablation that contained 5% nickel and 5% molybdenum, while studies by Shvedova et al and Lam et al used SWCNT synthesized by HiPCO that contained residual iron concentrations of 0.23% and 2.14%, respectively. Iron has been proposed to mediate the toxic effects of air pollution particles and asbestos fibers by generating reactive oxygen species through the Fenton reaction [15,16]. Moreover, loading nonfibrogenic titanium dioxide particles with iron has been shown to make these particles fibrogenic in a rat tracheal explant model [17]. Therefore, metals such as cobalt and iron likely contribute to the surface reactivity of SWCNT. This is supported by our observation that CB nanoparticles, which possessed the same surface area as SWCNT and yet did not contain contaminating metals, caused no fibrotic effects in the lungs of rats. Warheit and colleagues recently reported that various quartz particles produced differential degrees of pulmonary toxicity correlated with surface activity rather than particle size [18]. We further propose that low levels of contaminating metals coupled with high surface area determine the toxicity and fibrogenic potential of SWCNT.

We discovered unique carbon structures formed of SWCNT that bridged a small percent of alveolar macrophages within the lung. Approximately 5% of alveolar macrophages collected by BAL contained carbon inclusions after exposure to either CB or SWCNT. Approximately one fourth of the macrophages with carbon inclusions in the SWCNT-exposed rats were joined by carbon bridges at 21 days post-exposure. The unique hourglass structure of carbon bridges that joined macrophages were not readily apparent in BAL cytospins at day 1 post-SWCNT exposure, although some groups of macrophages were clustered around SWCNT aggregates thereby indicating the early stages of bridge formation. Carbon bridges were not observed in any CB-exposed rats despite similar numbers of lung macrophages that engulfed carbon aggregates composed of either CB and SWCNT in a comparable size range of 0.5 to 3 μm. The appearance of micron-sized carbon bridges between macrophages at 21 days was so reliable that we were able to correctly identify all SWCNT-exposed rats by evaluating the BAL cytospins using light microscopy in a blinded analysis. These structures may have important future implications for determining human exposure to SWCNT.

How carbon bridges form in the lung after SWCNT is not entirely clear. Preliminary observations with NR8383 macrophages indicate that some bridges form during cytokinesis with the resulting daughter cells joined by aggregated bundles of SWCNT (Mangum and Bonner, unpublished observation). We did not detect carbon bridges between macrophages *in situ *in the lungs of rats or in BAL cytospins 1 day after SWCNT exposure. This observation suggests that phagocytosis by two or more macrophages is an unlikely mechanism of bridge formation. In addition, bridge formation is not akin to "frustrated" or incomplete phagocytosis of long (>17 μm) asbestos or man-made fibers, where macrophages viewed in real time avidly move along the length of the fiber [19]. Furthermore, the biological or functional consequence of carbon bridge formation has yet to be determined. Carbon bridges could affect the phagocytic function of macrophages or inhibit their ability to release or respond to cytokines. While potentially important, these issues could be difficult to address as only about one percent of alveolar macrophages within the lung at 21 days post-exposure were linked by carbon bridges. Because of the low percentage of macrophages linked by carbon bridges, it is also not likely that these structures would have a deleterious effect on the overall lung macrophage population.

## Conclusion

Despite similar size and specific surface area of carbon black nanoparticles and SWCNT used in this study, only SWCNT caused 1) interstitial fibrotic lesions in the lungs of rats, 2) significant increases in lung PDGF mRNA levels, and 3) formation of unique carbon bridge structures between alveolar macrophages *in situ*. These differences between carbon black nanoparticles and SWCNT suggest that the fibrogenic activity of SWCNT is due to factors that make them unique from carbon black nanoparticles, including their unique shape or the presence of contaminating metal catalysts used in the manufacturing process. Finally, carbon bridge structures that form between macrophages are likely due to unique structural characteristics of SWCNT. Intercellular carbon bridge structures that link macrophages serve as a useful biomarker of SWCNT exposure in macrophages retrieved by bronchoalveolar lavage.

## Methods

### Animals

Six-week old female pathogen-free CDF (F344)/CrlBR rats were purchased from Charles River Breeding Laboratories, Kingston, NY, and housed in an International Association for Assessment and Accreditation of Laboratory Animal Care (AAALAC)-accredited humidity and temperature controlled facility. Rats were housed in microisolator cages on Alpha-dri cellulose bedding and supplied water and cereal-based diet NIH07 (Zeiger Brothers., Gardners, PA) ad libitum. The animal studies were approved by the CIIT Centers for Health Research Institutional Animal Care and Use Committee.

### General experimental design

The objective of this study was to determine whether SWCNT are fibrogenic in the lungs of rats after exposure by oropharygneal aspiration. Moreover, we sought to determine whether SWCNT increase mRNA levels of growth factors that mediate a fibrogenic response. We hypothesized that SWCNT would increase the expression of pro-fibrotic growth factors (PDGF, CTGF, TGF-β1) and thereby induce interstitial fibrosis. Oropharyngeal aspiration is a standard method for delivering particle suspensions to the lung and results in better particle distribution throughout the lung as compared to intratracheal instillation. Quantitative real time RT-PCR is the optimal method for measuring changes in growth factor mRNA levels in the lungs of exposed animals and changes in mRNA can be normalized for several standard housekeeping genes (described below under *Taqman quantitative real time RT-PCR*). Animals were acclimated for two weeks and then randomly assigned into treatment groups according to body weight prior to particle exposure. Rats were exposed to 2 mg/kg of SWCNT by oropharyngeal aspiration. Negative control animals were treated with Ca^2+ ^and Mg^2+ ^-free phosphate-buffered saline (PBS) with 0.1% pluronic. Carbon black (CB) particles were used as a negative control particle that does not cause fibrosis. Vanadium pentoxide (V_2_O_5_) was used as a positive control for causing a fibrotic response in the lungs of rats. Animals were killed by pentobarbitol overdose at 1 and 21 days following particle exposure. The lungs were lavaged with PBS. The left lungs were used to assess lung cell proliferation and histopathology. The right lung lobes were snap-frozen in liquid nitrogen stored at -80°C until used for RNA isolation and assessment of gene expression by real time quantitative RT-PCR.

### Particles

High-purity SWCNT were purchased from Helix Material Solutions, Richardson, TX [19]. These SWCNT were produced by a chemical vapor deposition process yielding particle external diameters of less than 2 nm with lengths ranging from 0.5 to 40 microns and a purity > 90% as determined by transmission electron microscopy (TEM), scanning electron microscopy (SEM), Raman spectroscopy and thermogravimetric analysis (TGA). SWCNT contained less than 5% amorphous carbon. The specific surface area of SWCNT was 300–600 m^2^/g. The elemental composition of SWCNT as determined by energy dispersive X-ray diffraction was 89.6% carbon, 6.1% oxygen, 2.6% cobalt (Co), and 1.7% molybdenum (Mo). Co and Mo were metal catalysts used in the chemical vapor deposition process. Nanosized CB particles (Raven 5000 Ultra II) were obtained from Columbian Chemicals Company, Marietta, GA [20]. The Raven 5000 Ultra II CB had a mean particle size of 8 nm and specific surface area between 350–583 m^2^/g. Vanadium pentoxide V_2_O_5_, (also referred to as vanadium oxide V) was obtained from Sigma-Aldrich, (St. Louis, MO). V_2_O_5 _was used as a positive profibrogenic agent and was purchased as an 8 micron mesh size (i.e., particles <8 micron). However, V_2_O_5 _is at least partly soluble in aqueous solutions and was therefore not compared on a size basis to CB and SWCNT. Dry CB and SWCNT were milled in a Retsch Mixer Mill (Retsch Inc., Newtown, PA) for 5 minutes at 30 cycles per second. The milled nanoparticles were then suspended in a biocompatible nonionic surfactant, 1% Pluronic F-68 (BASF Corp., Florham Park, NJ) in PBS and wet milled for an additional 5 min. The CB and SWCNT suspensions were further diluted with PBS to achieve the desired final dosing concentration of 0.1% Pluronic F-68. V_2_O_5 _particles were suspended in PBS with 1% Pluronic F-68, sonicated for 30 minutes, then further diluted 1:10 with PBS. Lastly, all particles were kept dispersed by placing suspensions in an ultrasonic waterbath until vortex mixed just prior to instillation. All particles were sterilized prior to instillation.

### Instillation of nanoparticles

Rats were administered particles by oropharyngeal aspiration. In brief, animals were anesthetized with isofluorane and ~100 μl volume of either CB, SWCNT or V_2_O_5 _(dose concentration equivalent of 2 mg of particles per kg of bodyweight) was placed at the back of the throat while holding the rat's tongue until the suspension was aspirated into the lungs. Control rats were administered an equivalent volume of PBS with 0.1% Pluronic F-68 surfactant.

### Bronchoalveolar Lavage

Rats were euthanized by pentobartital overdose and lungs were lavaged five times with 5-ml volumes of PBS. Two bronchoalveolar lavage (BAL) samples representing the first two and subsequent three recovered lavages were pooled and placed on ice. We established that this gentle lavage procedure does not compromise lung architecture as compared to animals that were not lavaged nor does it induce mRNA levels encoding pro-inflammatory cytokines or pro-fibrotic growth factors. BAL cells collected by centrifugation were resuspended in culture medium and enumerated using an automated cell counter (Model ZM, Coulter, Marietta, GA). Cytospins were prepared with approximately 10^5 ^cells per slide. Cell differential counts were performed on HEMA-3 (Fisher Scientific, Pittsburgh, PA) stained cytocentrifuge slide preparations. Total protein and LDH in cell-free BALF from the first two pooled lavages were analyzed spectrophotometrically using a COBAS FARA II (Roche Diagnostic Systems Inc., Montclair, NJ).

### Lung histopathology and cell proliferation

One hour prior to euthanasia, rats received a single intraperitoneal injection of 50 mg/kg body weight of bromodeoxyuridine (BrdU; Sigma-Aldrich). At necropsy, left lungs were pressure-infused intratracheally (30 cm H_2_O) with 10% neutral-buffered formalin. Lungs were fixed for approximately 48 h and then changed to 70% ethanol. Subsequently, the lungs were embedded in paraffin, sectioned at 5 μm, and stained with Masson's trichrome or immunostained for bromodeoxyuridine (BrdU) by established methods [22]. Cell labeling indices were determined in the bronchiolar/alveolar region and in the bronchus-associated lymphoid tissue for each animal, and the mean labeling index was calculated for each group of five animals.

### Cytology and carbon bridge assessment

In order to validate our observations in a quantitative, unbiased manner, BAL cell cytospin slides were scored in a blinded fashion; i.e., without knowing which cytospins were from CB or SWCNT exposure groups. Cell numbers were quantified by light microscopy using the 40× objective and a squared grid eyepiece graticule (field area = 0.173 mm^2^). Ten microscopic fields were counted per animal and at least 500 cells per animal were counted. Four parameters were measured; A) Total cells/field, B) Numbers of cells per field containing carbon inclusions, C) Carbon bridges between macrophages per field, and D) Mitotic figures per field.

### Taqman quantitative real time RT-PCR

Total RNA from right anterior lungs of particle-treated rats was isolated using TRIZOL reagent (Invitrogen, Carlsbad, CA), followed by RNA cleanup performed using RNeasy Midi spin columns (Qiagen, Valencia, CA). One or two micrograms of total RNA was reverse transcribed at 48°C for 30 minutes using Moloney murine leukemia virus reverse transcriptase (Eurogentec, San Diego, CA) in 1 × RT buffer, 5 mM MgCl_2_, 500 μM of each dNTP, 2.5 μM of random nonamers, and 0.4 U/μL RNase inhibitor in a volume of 100 μl. Twenty nanograms of the RT product was amplified using Taqman Gene Expression Assays specific for platelet-derived growth factor receptor alpha (PDGFRα), PDGF-A, PDGF-B, PDGF-C, transforming growth factor beta-1 (TGF-β1), osteopontin (OPN), connective tissue growth factor (CTGF), Type I procollagen (COL1A2), and 18S on the Applied Biosystems 7900 Prism^® ^Sequence Detection System (Applied Biosytems, Foster City, CA). The PCR conditions and data analysis were performed according to the manufacturer's protocol described in User bulletin no.2, Applied Biosystems Prism 7700 Sequence Detection System. Gene expression was measured by the quantitation of cDNA converted from mRNA corresponding to the target genes relative to the vehicle-treated control groups and normalized to eukaryotic 18S reference endogenous control. Relative quantitation values (2^-ΔΔCT^) were expressed as fold-change over controls.

### Statistical methods

All data were tested for normality and homogeneity of variance. Gene expression data were log transformed and comparisons to controls were made using Dunett's test (P < 0.05). The software package JMP (SAS Institute, Cary, NC) was used for the statistical analysis.

## Abbreviations

SWCNT, single-walled carbon nanotubes; CB, carbon black; V_2_O_5_, vanadium pentoxide; PDGF, platelet-derived growth factor; OPN, osteopontin; CTGF, connective tissue growth factor; TGF-β1, transforming growth factor-β1.

## Competing interests

The author(s) declare that they have no competing interests.

## Authors' contributions

JCB and JBM had the initial idea of performing the studies. JCB designed the experiments with input from JBM, EAT, AAM, and EB. Exposures of rats to particles and necropsy was performed by JBM, EAT, and EB. The original discovery of carbon bridges between macrophages from the lungs of rats exposed to nanotubes was made by JBM and then quantified in a blinded assessment by JCB. All RT-PCR was performed by JBM. Pathology was performed by MFC and JCB. All authors read, reviewed and approved all versions of the manuscript.

## Acknowledgements

We thank Carol Bobbitt, David Weil, and Victoria Wong for excellent technical expertise. We are also grateful to Dr. David Dorman for invaluable comments during the preparation of this manuscript. This work was funded by The American Chemistry Council's Long Range Research Initiative provided to CIIT Centers for Health Research.
